# Use of a hexapod in diffraction measurements of substrate-supported crystals of organic semiconductors

**DOI:** 10.1107/S0909049509037911

**Published:** 2009-10-14

**Authors:** Lin Yang, Hoichang Yang

**Affiliations:** a National Synchrotron Light Source, Brookhaven National Laboratory, USA; b Department of Advanced Fiber Engineering, Inha University, Korea

**Keywords:** X-ray diffraction, hexapod, transmission, grazing incidence

## Abstract

Thin films of organic semiconductor prepared on substrates generally contain crystals that have one common crystal plane parallel to the substrate but random in-plane orientations. In diffraction measurements of these structures, it is often required to anchor the X-ray beam on a fixed spot on the sample, such as an optically visible crystallite or island. Here, a hexapod is used in place of a traditional multi-circle diffractometer to perform area-detector-based diffraction measurements on an actual device that contains 6,13-bis(triisopropyl­silyethynyl)-pentacene (TIPS-pentacene) crystals. The hexapod allows for sample rotations about any user-defined rotation center. Two types of complex sample motions have been programmed to characterize the structure of the TIPS-pentacene crystal: an in-plane powder average has been performed at a fixed grazing-incident angle to determine the lattice parameters of the crystal; then the in-plane component of the scattering vector was continuously rotated in transmission geometry to determine the local crystal orientation.

## Abstract 2

The use of a hexapod in X-ray diffraction measurements of substrate-supported TIPS-pentacene crystals has been demonstrated to determine the lattice constants and the local orientation of the crystals.

## 1. Introduction

There has been a growing interest in developing organic semiconductor materials for applications such as organic field-effect transistors (OFETs), organic light-emitting diodes and organic photovoltaic devices (Dimitrakopoulos & Malenfant, 2002 ▶). The charge transport properties of organic semiconductors are determined by the spatial arrangement of molecular orbitals within the material. An important step in the development of these materials is therefore to determine the crystalline structure of the material within the device and understand how it is correlated to the device performance. Since these devices are often fabricated on a flat substrate, the crystal structure can be characterized by grazing-incidence X-ray diffraction (GID) measurements. While GID measurements on substrate-supported organic semiconductors have been traditionally carried out using point detectors (*e.g.* Fritz *et al.*, 2004 ▶; Ruiz *et al.*, 2004 ▶; Yoshida & Sato, 2006 ▶), recent studies have employed area detectors to reduce the time required to collect the full diffraction pattern and therefore the radiation damage to the sample (Yang *et al.*, 2005 ▶). GID measurements that utilized area detectors have also utilized X-ray beams that are focused in the direction of the sample normal to reduce the beam footprint on the sample (Yang, 2005 ▶), so as to achieve angular resolution comparable to those in point detector-based measurements.

The same X-ray method is also used in studies of other substrate-supported layered structures, such as model biological lipid membranes. Technical details have already been worked out to translate the two-dimensional GID pattern into an undistorted *q*_*r*_–*q*_*z*_ map (*q*_*r*_ and *q*_*z*_ are components of scattering vectors parallel and perpendicular to the substrate, respectively) and to correct integrated diffraction intensities for the purpose of structural determination (Yang *et al.*, 1998 ▶; Yang & Huang, 2003 ▶). In these measurements, an almost complete diffraction pattern can be obtained in a single X-ray diffraction pattern with fixed X-ray incident angle, provided that the crystals being probed have random in-plane orientations, or if the sample is rotated about the substrate normal during the measurement to artificially create an in-plane powder. The missing information on layer spacing can be obtained from an additional diffraction pattern in which the sample rotation is varied continuously during the exposure so that the Bragg condition can be sequentially satisfied for the layer peaks during sample rotation, comparable to an X-ray reflectivity measurement.

As solution-based processes are being explored to reduce production costs (Forrest, 2004 ▶), the thin films of small-molecule organic semiconductors prepared using these processes often contain single-crystalline domains that are tens, or even hundreds, of micrometers wide and larger than the dimension of an individual device. It is of great interest to carry out separate diffraction measurements within each individual device and examine the correlation between device performance and the crystal orientation. In practice, this requires the rotation center to be redefined during a series of diffraction measurements. While redefining the rotation center on the sample is challenging for a traditional multi-circle diffractometer, it can be easily accomplished by a hexapod. In this paper, we will explore the use of the hexapod in X-ray diffraction measurements on the TIPS-pentacene crystal in an actual device. We will perform in-plane rotation in GID measurement to determine the TIPS-pentacene crystal lattice constants and determine the in-plane orientation of the crystalline domain within the device using transmission diffraction.

## 2. Experimental methods

### 2.1. Experimental set-up

**Figure 1:**
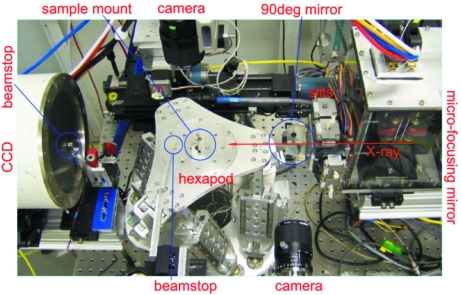
Experimental set-up used for the diffraction measurements. In the actual measurements a plastic bag filled with helium was used to cover the hexapod in order to reduce background scattering.

The X-ray diffraction measurements were carried out at beamline X21 of NSLS (Yang, 2005 ▶) using the set-up shown in Fig. 1 ▶. Diffraction patterns were recorded on a Mar 165 CCD detector (165 mm diameter, 1024 × 1024 pixels). The sample-to-detector distance was chosen to be ∼20 cm and the X-ray energy was 13 keV so that the detector captures diffraction peaks at up to *q*_*r*_ ≃ 3.1 Å^−1^. In order to reduce the X-ray footprint on the sample and thus improve angular resolution in GID measurements, the X-ray beam was focused in the vertical direction to ∼15 µm FWHM at the sample position by a 10 cm-long micro-focusing mirror (XRadia). The X-ray footprint is therefore ∼0.9 mm long when the incident angle is 1°. The horizontal beam size was ∼50 µm as defined by slits. In order to visualize the part of the sample being probed, two cameras were used: one looking at the sample from above, and the other looking along the beam using a mirror positioned at 45° from the beam and with a clear aperture to allow the X-ray beam to pass through. Two different beam stops were used: one just upstream of the detector with an embedded photodiode for sample alignment in grazing-incidence geometry; the other, located close to the sample, was only used in diffraction measurements in transmission geometry to stop the direct beam immediately after the sample and reduce background scattering.

### 2.2. Organic semiconductor device

As a demonstration of the method, we will report results of measurements on a TIPS-pentacence test device fabricated for the purpose of carrier mobility characterization. The semiconducting material was first deposited onto a piranha-cleaned SiO_2_ (300 nm thick)/heavily doped Si substrate (2 cm × 2 cm) *via* drop casting from a 0.25 wt% solution of TIPS-pentacence in toluene. Top-contact gold source and drain electrodes were then vacuum-deposited onto the TIPS-pentacene crystals through a shadow mask. The electrodes are 800 µm wide and the gap between the two electrodes is 100 µm [see Fig. 9(*c*) for a photograph of the device]. In the carrier mobility measurement, voltages were applied between the electrodes. The carrier mobility for the material was then calculated from the drain-source current *versus* gate (substrate) voltage transfer curves of the OFET device operated in a well defined saturation regime (see, for example, Yang *et al.*, 2007 ▶).

### 2.3. Hexapod

The hexapod (ALIO Industries, model AL-HEX-HR4) was mounted on a full-circle rotary stage in order to realise 360° in-plane rotation for the sample. Both the hexapod and the rotary stage are equipped with NanoMotion ceramic motors and Renishaw encoders. The motors were commanded by an SPiiPlus SA-8 (ACS Motion Control) motion controller, which in turn communicated to the beamline control software *SPEC* (Certified Scientific Software) through an ethernet link.

**Figure 2:**
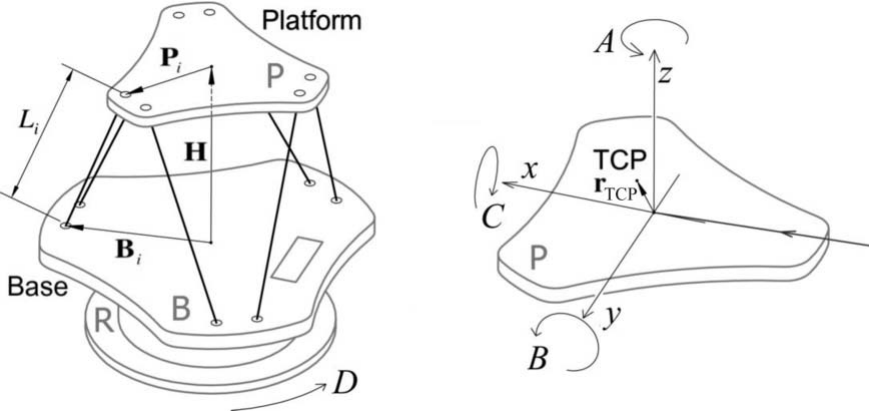
Geometry of the hexapod and definition of the vectors and axes involved in the kinematics calculations. The user-defined rotation center is denoted TCP, which is initially set at the center of the hexapod platform.

The ACS controller was shipped with kinematics codes (see SPiiPlus documentation at http://www.acsmotioncontrol.com/) that translate between physical motor positions and six logical axes: the rotations *A* (yaw), *B* (pitch) and *C* (roll) of the hexapod platform and its translations *x*, *y* and *z* (Fig. 2 ▶). Below, we first review the basic principle of the kinematics calculations implemented in these codes in §2.3.1. We then discuss in the subsequent subsections the specific requirements for using the hexapod in diffraction measurements and the necessary revisions to the kinematics codes.

#### 2.3.1. Basic kinematics calculations

The inverse kinematics (IK) code calculates the physical motor position in the six actuators, {*m*_*i*_}, from axis positions {*a*_*i*_} = {*A*, *B*, *C*, *x*, *y*, *z*}; while the forward kinematics (FK) calculation finds {*a*_*i*_} for a given set of {*m*_*i*_}. The FK calculation cannot be expressed in a simple analytical form. Instead the FK code is implemented as an iterative search that resorts to the IK calculation. The IK code utilizes the pitch–roll–yaw rotation matrix *R*(*A*, *B*, *C*) to calculate the absolute position of the joints on the top platform of the hexapod from the position of the rotation center and the position of the joints, all relative to the center of the top platform, which can be defined by the user,
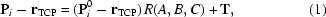
where **T** = (*X*, *Y*, *Z*) is the translation required of the rotation center, and the rotation matrix is defined as
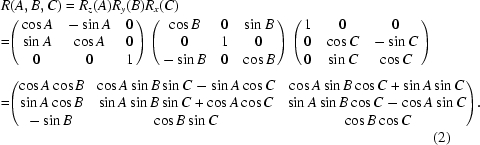
The lengths of the six legs and the physical motor positions are then calculated from the positions of the joints on the top and bottom platforms,
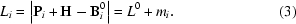
Here, **P** and **B** are the vectors pointing from the center of the platform and the base, respectively, to the joint for the *i*th leg on the hexapod, and **H** is the vector connecting the two centers, as defined in Fig. 2 ▶.

#### 2.3.2. Hexapod alignment

The goal of the alignment is to ensure that the X-ray beam passes through the rotation center defined by the user. Whereas alignment of a conventional diffractometer requires physical adjustments of the instrument, alignment in this case is realised by modifying the kinematics codes in the controller and adjustments are made continuously depending on the position of the hexapod.

**Figure 3:**
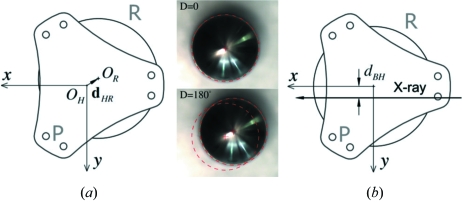
Schematics that illustrate alignment of the centers of the hexapod (*O*_H_) and the rotary stage (*O*_R_). Alignment requires finding both (*a*) offset between *O*_R_ and *O*_H_, and (*b*) once they coincide, the distance from the common rotation center to the X-ray beam. The offset can be measured directly from images capture using the camera overlooking a 0.25′′ tooling ball used to indicate the center of the hexapod platform. Each pixel in the image corresponds to ∼10 µm. The measured offset is (Δ*x*_HR_, Δ*y*_HR_) = (0.54 mm, 0.75 mm).

The alignment procedure must first compensate for the offset between the center of the hexapod platform and the axis of the rotary stage, *d*_HR_ = (Δ*x*_HR_, Δ*y*_HR_). In order for the two to coincide, the kinematics calculation needs to include a correction term for **P**_*i*_,
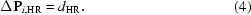
The value of this offset can be found by simply rotating the rotary stage by 180°. The apparent displacement of the center of the hexapod platform, as indicated by a 1/4-inch tooling ball in the view *via* the camera overlooking the hexapod from above (Fig. 3*a* ▶), equals 2*d*_HR_. 

In general there is also an offset, *d*_BH_, between these common rotation centers and the X-ray beam. The kinematics codes compensate for this offset by moving the hexapod by the same distance towards the beam to position the hexapod rotation center into the X-ray beam. This compensation is dependent on the position of the rotary stage and is accounted for in the kinematics calculation by another correction term,
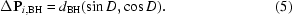
The actual value of *d*_BH_ can be found by scanning the *y*-position of a sharp edge with known distance from the hexapod platform center (*e.g.* the edge of the tooling ball shown in Fig. 3*a* ▶) and using the photodiode embedded in the beam stop as the detector (this will be implicitly assumed in the description below). The correction above also requires the zero position of *D* to be defined as the position where the *x*-axis is parallel to the X-ray beam. This can be done again by *y*-scanning the sharp edge at two extreme *x* positions. The two scans should produce the same results when *D* = 0 is correctly defined.

Next the rotation center of the hexapod must be positioned at the same height as the X-ray beam. The offset between the hexapod rotation center and the beam position, *d*_*z*_, can be found using the top edge of the tooling ball, the distance from which to the hexapod rotation center is known. The default distance between the hexapod platform and the base is then revised, *H* = *H*_0_ + *d*_*z*_, so that the default position of the hexapod rotation center is now located in the path of the X-ray beam.

It is important to note that whenever any of the values (logical motor position, rotation center position and correction terms) that enter the kinematics calculations are revised, the nominal position of the hexapod, **T**, must be revised accordingly so that the outcome of the calculations, *i.e.* the positions of the actuators, remain the same. Furthermore, the revision of these values must be completed within one single controller cycle. Failure to do so will result in a critical motion error by the motion controller as it attempts to maintain actuator positions calculated from the revised kinematics calculations.

#### 2.3.3. Redefining the rotation center

The hexapod kinematics codes in principle allow any arbitrary point to be defined as the rotation center. At the end of the initial alignment as described in §2.1, the hexapod rotation center is in the path of the X-ray beam. This rotation center is visualized in the views from the two cameras and used as a reference to move the part of the sample to be measured into the X-ray beam. The current position can then be redefined as the new TCP and the value of **T** is reset to zero. The new value of **r**_TCP_ can be found *via* the requirement that the physical positions of the hexapod joints remain the same before and after the redefinition of TCP,
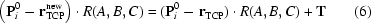
or
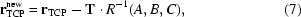
where *R* ^−1^(*A*, *B*, *C*) is the inverse of, and simply transposed from, the current pitch–roll–yaw matrix.

#### 2.3.4. Sample alignment

Angular alignment must be performed with actual samples since the sample substrate may not necessarily be parallel to the hexapod platform or the sample support.

For grazing-incidence measurements, the zero position of *A* does not need to be redefined since the alignment procedure of *D* described in §2.3.2 implicitly assumes that *A* is already aligned, *i.e.* the *x*-axis is parallel to the X-ray beam. Once the rotation center is already defined on the sample surface, alignment of *B* can be accomplished utilizing the CCD detector. At *D* = 0, the X-ray beam is reflected by the sample at incident angle −*B*_0_ and the specular reflection is recorded on the CCD. The hexapod is then turned by 180° to *D* = 180°. The specular reflection from the sample is again recorded, but at *B* = *B*_0_. The two reflections should coincide if the zero for *B* is correctly defined.

**Figure 4:**
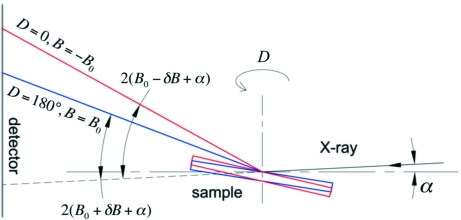
Illustration of angles involved in the alignment of hexapod pitch, *B*, in grazing-incident geometry.

In reality, there is a finite offset of the nominal zero position from the true zero position, δ*B*. There is also a slight downward angle, α, between the incident X-ray beam and the horizontal plane (hexapod *XY* motion) owing to the vertical focusing mirror. The two reflections are therefore split (Fig. 4 ▶). Once the pixel positions on the detector are calibrated with a standard sample, the angle between the reflected beam and the direct beam can be found from the corresponding *q* value. The values of α and δ*B* therefore can be solved. *C* can be aligned similarly at *D* = 90° and −90°. The accuracy of these alignment procedures is limited by the scattering angle that corresponds to the half-width of one detector pixel, which is ∼0.02° in our measurements.

In transmission geometry, *A* and *B* must be aligned so that the sample is perpendicular to the X-ray beam at *A* = *B* = 0. The intensity observed by the beam-stop photodiode is used as a guide. *A* can be aligned as follows. At *D* = 90°, half-cut the observed X-ray beam intensity with the sample by adjusting the sample *x* position. The intensity on the photodiode is then monitored during an *A*-scan. The zero position of *A* corresponds to the center position of the scan, or the maximum intensity detected by the photodiode. *B* can be aligned by scanning *x* with *A* = 0, *D* = 90°, and at two extreme positions of *z* (top and bottom of the sample). The two scans should yield the same results if *B* is correctly aligned. In both cases, the alignment accuracy is limited by a fraction of the horizontal beam size (∼1/5 of 50 µm) and the sample size (>1 cm). We estimate it to be ∼0.05°.

## 3. Results and discussions

To illustrate how the hexapod is used in actual measurements, we present below experimental data collected from the actual device described in §2.2. We extract the lattice parameter of the TIPS-pentacene crystal from the GID data. We then examine the local crystal orientation within the single-crystalline domains located between the source and drain gold pads.

### 3.1. Crystal lattice parameter determination using GID

As discussed in the *Introduction* [Sec sec1], the GID pattern from the sample should be recorded while the sample is rotated about the substrate normal to create an in-plane powder average. In this process the X-ray incident angle onto the substrate needs to be kept constant by virtue of concerted motion of both the rotary stage and the hexapod.

**Figure 5:**
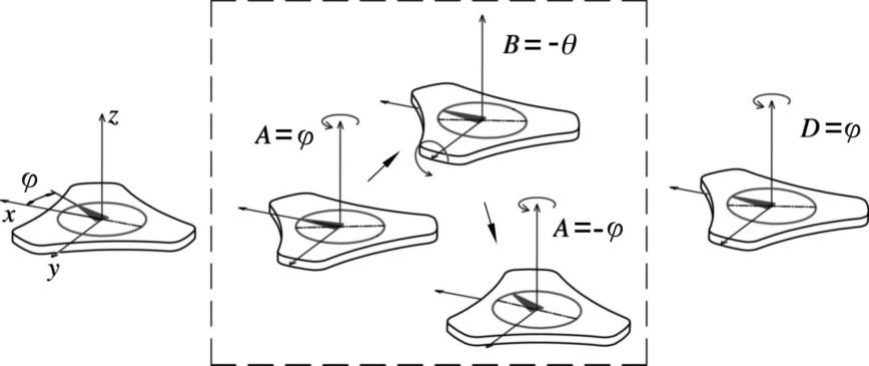
Illustration of motions required for achieving arbitrary in-plane crystal orientation ϕ at a given incident angle θ. The objective is to align the interested in-plane vector to the projection of the incident beam. The three steps in the dashed box are compounded into one equivalent hexapod motion.

Assume that the in-plane orientation that corresponds to azimuthal angle −ϕ is to be positioned along the incident beam in the GID measurement (Fig. 5 ▶). The sample must be rotated by *A* = ϕ followed by another rotation of *B* = −θ to achieve the desired incident angle. However, because of the hexapod’s limited yaw travel (∼±15°), this may not be physically feasible. Therefore we follow this with mutually cancelling rotations of *A* = −ϕ by the hexapod and *D* = ϕ by the rotary stage. The three rotations by the hexapod (enclosed by the dashed box in Fig. 5 ▶), when combined, amount to equivalent but small yaw, roll and pitch motions within travel ranges. The overall motion for arbitrary azimuthal angle ϕ is therefore now always within the travel ranges of hexapod rotations.

**Figure 6:**
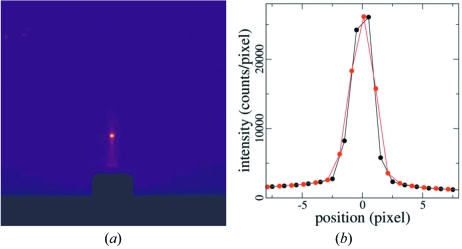
(*a*) CCD image of a specular reflection from a polystyrene (10 nm thick) coated Si substrate at 1.5° incident angle and with full-circle in-plane rotation. The sample normal points up. (*b*) Comparison of the vertical intensity profiles of the specular peak with (red) and without (black) in-plane motion.

The rotation matrix corresponding to the combined hexapod rotations is
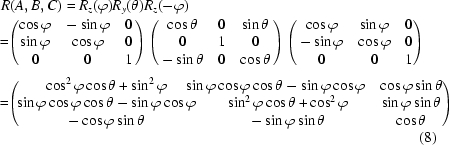
with equivalent rotations
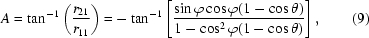

               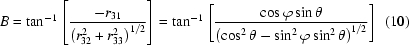
and
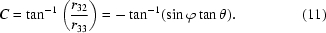
The accuracy of the incident angle during the in-plane sample rotation is illustrated by the stability of the specular reflection from the sample. Fig. 6 ▶ shows a CCD image of the specular reflection from a bare silicon substrate during a full-circle in-plane rotation. The specular peak remains sharp and its width is essentially identical to that recorded without sample rotation, showing that the incident angle is much more accurate than the reflection angle that corresponds to the pixel width, which is ∼0.04°. This accuracy is mainly limited by the accuracy of the alignment of hexapod pitch and yaw angles.

**Figure 7:**
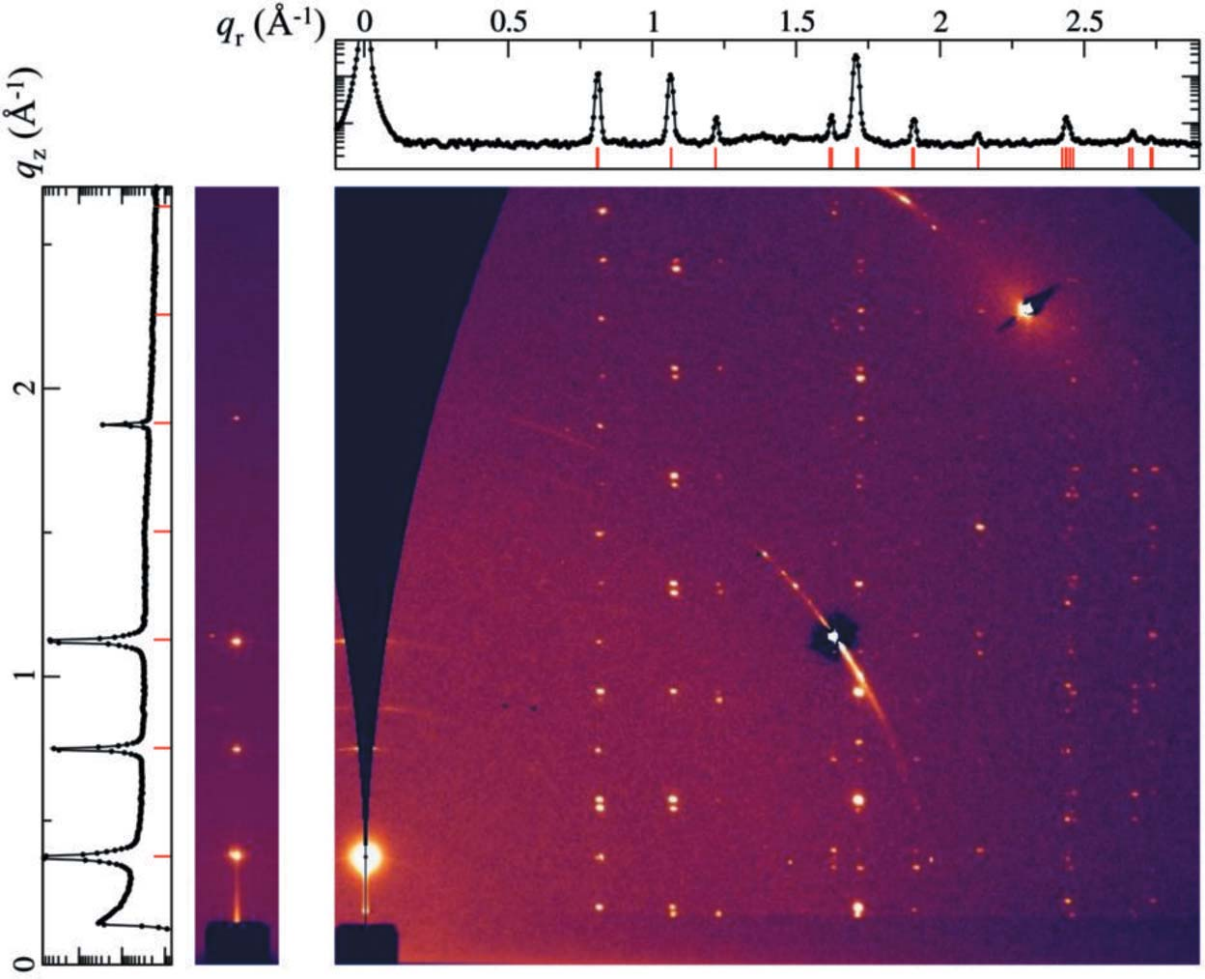
Grazing-incidence diffraction data collected from the TIPS-pentacene sample. On the left is the CCD image collected with continuously varying incident angle and the intensity profile along the (00*L*) direction. On the right are the CCD images collected at 1.5° incident angle with continuous in-plane sample rotation and the intensity profile collapsed onto the *q*_*r*_ axis. Note that the CCD image on the right has been translated onto the undistorted *q*_*r*_–*q*_*z*_ plane, hence the dark gap in the upper left corner, which corresponds to information not accessible in the diffraction pattern. A diffraction pattern from a bare silicon substrate was subtracted as scattering background.

The GID data collected from the sample are shown in Fig. 7 ▶. A grazing-incidence diffraction pattern (right) was first recorded with full-circle in-plane sample rotation during data collection. The incident angle was chosen to be 1.5° in order to limit the beam footprint on the sample (∼0.6 mm) and therefore maintain the angular resolution in the data at high *q*. This diffraction pattern contains most of the diffraction peaks except for those located near the *q*_*z*_ axis since the Bragg condition cannot be satisfied for these **q** values, reflecting the curvature of the Ewald sphere. The reflections with small *q*_*z*_ values are blocked by the substrate. A second diffraction pattern (left) was then recorded without in-plane rotation but the incident angle was varied continuously during data collection. The Bragg peaks that correspond to the stacking of the crystal plane that are parallel to the substrate are recorded in this pattern when the Bragg condition is satisfied for each (00*L*) peak during the rotation of the incident angle. The positions of these peaks give the layer spacing *d* = 16.71 ± 0.04 Å and the corresponding reciprocal vector c* = 0.376 ± 0.001 Å^−1^.

For a substrate-supported crystal, the reciprocal vector, c*, is perpendicular to the substrate. By choosing the direction of the in-plane projection of a* to be the *q*_*x*_-axis, the reciprocal vectors can be written as
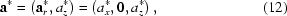

               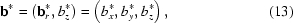

               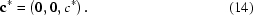
The observed diffraction peaks are therefore located on columns (*HK*) with constant **q**_*r*_ = 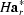 + 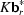. Once the GID pattern at constant incident angle was converted to an intensity map on the *q*_*r*_–*q*_*z*_ plane (Fig. 7 ▶), the *q*_*r*_ positions of these columns were extracted and indexed (see Table 1 ▶) to a two-dimension lattice 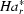 = 0.812 ± 0.007 Å^−1^, 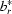 = 0.808 ± 0.001 Å^−1^ and γ_*r*_ = 82.3 ± 0.3°. The (*HK*) index of each column and the *q*_*z*_ positions of the peaks within the column were then combined to give *a*_*z*_ = 0.173 ± 0.001 Å^−1^ and *b*_*z*_ = −0.003 ± 0.001 Å^−1^. The final lattice constants of the crystal are therefore *a* = 7.81 ± 0.07 Å, *b* = 7.85 ± 0.01 Å, *c* = 17.09 ± 0.05 Å, α = 88.2 ± 0.2°, β = 102.2 ± 0.1°, γ = 97.7 ± 0.3°. This is a different structure than those reported by Chen *et al.* (2007 ▶) (*a* = 7.55 Å, *b* = 7.73 Å, *c* = 16.76 Å, α = 89.5°, β = 78.7°, γ = 84.0°) and Kim *et al.* (2007 ▶) (*a* = 7.57 Å, *b =* 7.75 Å, *c* = 16.84 Å, α = 89.2°, β = 92.7°, γ = 83.6°). This kind of polymorphism is common for small-molecule organic semiconductors. The existence of polymorphism highlights the importance of structural characterization of materials in the actual device in order to truly understand its structure–performance relationship.

### 3.2. Crystal in-plane orientation determined by transmission diffraction

The transport properties of crystalline organic semiconductors are expected to be anisotropic. For instance, in a study of hole transport along pentacene crystals, Troisi & Orlandi (2005 ▶) reported distinct electron band dispersion along two mutually orthogonal in-plane orientations. Unlike other small organic semiconductor crystals grown from solution, TIPS-pentacene tends to grow large anisotropic crystals under slow solvent evaporation. It is therefore of particular interest to determine the orientation of the TIPS-pentacene crystal in actual devices.

**Figure 8:**
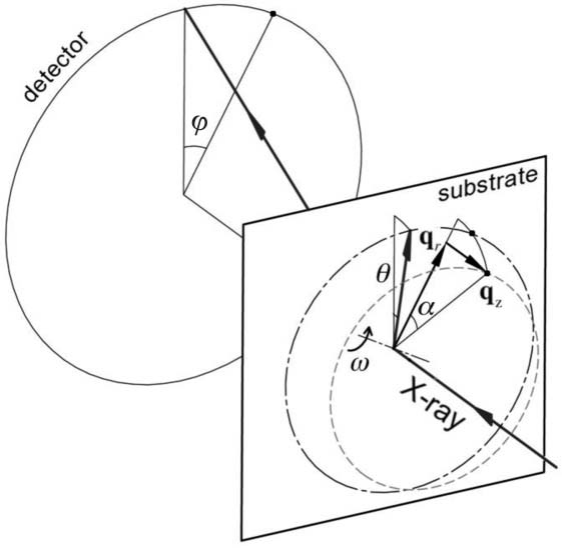
Geometry involved in detection of a Bragg peak at **q**_*HKL*_ = (**q**_*r*_, *q*_*z*_) in transmission geometry. In this illustration the sample is oriented perpendicular to the hexapod platform and the incident X-ray beam. For a given **q**_*HKL*_, the Bragg peak can only be located on the circle on the detector that corresponds to *q* = |**q**_*HKL*_|. The dash-dotted circle represents the trace on the Ewald sphere that corresponds to *q* = |**q**_*HKL*_|. The dashed circle represents the possible location of the **q**_*HKL*_ vector for the crystal being examined. The crystal must be rotated by ω = θ − α in the plane defined by the incident beam and **q**_*HKL*_, so that the reciprocal vector **q**_*HKL*_ for the crystal becomes located on the Ewald sphere and therefore the corresponding Bragg peak is recorded in the diffraction pattern.

While characterization of in-plane crystal orientation is possible using GID, the beam footprint in grazing-incident geometry is inevitably quite large owing to the small incident angle, compared with typical device size. Furthermore, a series of diffraction patterns that correspond to different in-plane orientations need to be recorded to identify the crystal orientation. Here, we utilize diffraction measurements in transmission geometry to determine the in-plane crystal orientation in the TIPS-pentacene samples in a single diffraction pattern, as illustrated in Fig. 8 ▶.

The orientation of the crystal can be represented by a single reciprocal vector **q**_*HKL*_ = (**q**_*r*_, *q*_*z*_). The in-plane orientation of **q**_*r*_, and therefore the crystal, can be determined if the corresponding diffraction peak is visible in the diffraction pattern, which requires the reciprocal vector to be located on the Ewald sphere. In turn, the crystal must be rotated, in the plane defined by **q**_*HKL*_ and the incident beam, by ω = θ − α, so that the diffraction peak is recorded at the azimuthal angle ϕ that corresponds to the orientation of **q**_*r*_. The actual value of ϕ, undetermined prior to the measurement, can be found by exhausting all possible values while the diffraction pattern is being recorded. The diffraction peak only appears when the ϕ angle at which the sample is turned coincides with the actual orientation of the crystal.

**Figure 9:**
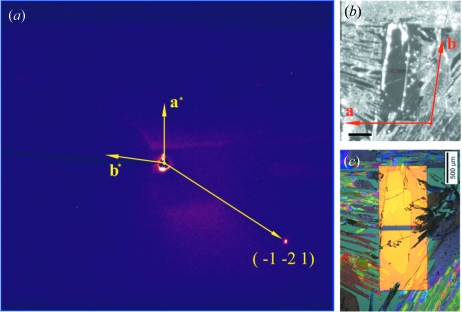
Diffraction data collected from the sample in transmission geometry and the inferred lattice orientation. The location where the diffraction pattern shown in (*a*) was collected is indicated by the red spot in (*b*), which is the view of the sample as seen from the camera *via* the 90° mirror. The scale bar is 0.5 mm. A higher-quality image observed by a polarized microscope is shown in (*c*) for comparison. Only one diffraction peak was observed in (*a*), indicating the single-crystalline nature of the sample. The orientation of the in-plane projection of the reciprocal vectors, as well as that of the unit vectors **a** and **b**, can be inferred from the location of the (−1 −2 1) peak, as shown in (*a*) and (*b*).

Sample rotation in the direction defined by ϕ as shown in Fig. 8 ▶ can be achieved as follows. The hexapod must first be rotated by a roll motion of *C* = −ϕ to orient **q**_*r*_ along the *z*-axis (pointing up in Fig. 8 ▶). The sample tilt is then achieved by a pure pitch motion of *B* = ω. The sample is finally returned to its original in-plane orientation by a hexapod yaw motion of *C* = ϕ. Again, these three motions can be compounded into one single hexapod motion represented by rotation matrix
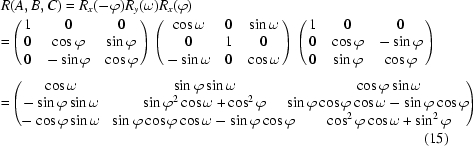
with equivalent rotations
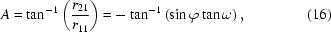

               
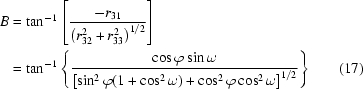
and
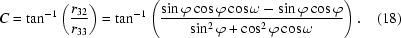
In practice, this diffraction peak selected to indicate the crystal orientation should have considerable intensity. This is because the material being examined can be quite thin (tens of nanometers), and therefore the diffraction peak can be overwhelmed by background scattering. The required sample rotations should also be minimal, owing to the limited hexapod rotation ranges. In our measurement, we selected the (−1 −2 1) peak, corresponding to *q* = 1.722 Å^−1^ (*q*_*r*_ = −1.709 Å^−1^, *q*_*z*_ = 0.208 Å^−1^) and ω = 0.35°. The X-ray beam passed through the sample between the two gold electrodes. The obtained diffraction pattern is shown in Fig. 9(*a*) ▶.

The in-plane projection of the reciprocal vector that corresponds to the (−1 −2 1) peak is given by *q*_*x*_ = −1.029 Å^−1^, *q*_*y*_ = −1.602 Å^−1^. Based on the location of the observed (−1 −2 1) peak, the orientation of the in-plane projection of the reciprocal vectors 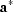 and 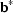 can therefore be established. In turn, the orientation of the unit vectors **a** and **b** can also be determined, as shown in Fig. 9(*b*) ▶. They are consistent with the morphology of the crystal.

## 4. Conclusions

We have demonstrated the use of a hexapod in X-ray diffraction measurements of substrate-supported TIPS-pentacene crystals to determine the lattice constants and the local orientation of the crystals. These measurements certainly can be performed using a conventional diffractometer as well. However, the hexapod has the major advantage that the user can arbitrarily define the rotation center. This is particularly useful when the sample contains multiple parts that need to be examined individually, such as the organic semiconductor device examined in this study.

The kinematics codes in the motion controller and the encoders in principle provide very high motion accuracy. The precision of the encoder for each actuator that connects the base and the platform is 2.44 nm count^−1^ and the maximum position error allowed by the motion controller is 80 counts, *i.e.* the precision of the length of the actuator, *L*_*i*_, is ∼0.2 µm. We therefore expect a precision of the same order of magnitude for the translational motions and ∼1 µrad for the rotations. The actual accuracy of the hexapod motion is also limited by the mechanical performance of joints between the platform/base and legs (Hephaist-Seiko SRJ-008C spherical rolling joints, 2.5 µm backlash error according to specification) and the physical dimensions of the hexapod that enter the kinematics codes. Characterization of these features of the hexapod is beyond the scope of this study.

## Figures and Tables

**Table 1:** Observed and expected *q*_*r*_ positions of diffraction peaks from TIPS-pentacene crystals Note that some lines are indistinguishable in the collapsed one-dimensional intensity *versus* *q*_*r*_ plot in Fig. 7 ▶; however, they clearly have different *q*_*r*_ values from the two-dimensional *q*_*r*_–*q*_*z*_ intensity map.

	*q*_*r*_ (Å^−1^)						*q*_*r*_ (Å^−1^)	
(*HK*)	Observed	Expected	(*HK*)	Observed	Expected
(0 1)	0.806	0.808	(2 2)	2.133	2.132
(1 0)	0.811	0.812	(0 3)	2.421	2.424
(1 1)	1.063	1.066	(3 0)	2.435	2.436
(1 −1)	1.223	1.220	(2 −2)	2.445	2.440
(0 2)	1.614	1.616	(1 3)	2.451	2.451
(2 0)	1.625	1.624	(3 1)	2.458	2.462
(1 2)	1.709	1.709	(1 −3)	2.652	2.658
(2 1)	1.712	1.714	(3 −1)	2.668	2.667
(1 −2)	1.902	1.903	(2 3)	2.730	2.731
(2 −1)	1.909	1.908	(3 2)	2.733	2.737
